# Analysis of SARS-CoV-2 viral loads in stool samples and nasopharyngeal swabs from COVID-19 patients in the United Arab Emirates

**DOI:** 10.1371/journal.pone.0274961

**Published:** 2022-09-22

**Authors:** Mariane Daou, Hussein Kannout, Mariam Khalili, Mohamed Almarei, Mohamed Alhashami, Zainab Alhalwachi, Fatima Alshamsi, Mohammad Tahseen Al Bataineh, Mohd Azzam Kayasseh, Abdulmajeed Al Khajeh, Shadi W. Hasan, Guan K. Tay, Samuel F. Feng, Dymitr Ruta, Ahmed F. Yousef, Habiba S. Alsafar, on behalf of the UAE COVID-19 Collaborative Partnership

**Affiliations:** 1 Department of Biology, Khalifa University of Science and Technology, Abu Dhabi, United Arab Emirates; 2 Center for Biotechnology, Khalifa University of Science and Technology, Abu Dhabi, United Arab Emirates; 3 Department of Biomedical Engineering, Khalifa University of Science and Technology, Abu Dhabi, United Arab Emirates; 4 Sharjah Institute for Medical Research, University of Sharjah, Sharjah, United Arab Emirates; 5 Department of Clinical Sciences, College of Medicine, University of Sharjah, Sharjah, United Arab Emirates; 6 Emirates Specialty Hospital, Dubai Healthcare City, Dubai, United Arab Emirates; 7 Medical Education and Research Department, Dubai Health Authority, Dubai, United Arab Emirates; 8 Center for Membranes and Advanced Water Technology (CMAT), Department of Chemical Engineering, Khalifa University of Science and Technology, Abu Dhabi, United Arab Emirates; 9 Division of Psychiatry, Faculty of Health and Medical Sciences, the University of Western Australia, Crawley, Western Australia, Australia; 10 School of Medical and Health Sciences, Edith Cowan University, Joondalup, Western Australia, Australia; 11 Department of Mathematics, Khalifa University of Science and Technology, Abu Dhabi, United Arab Emirates; 12 EBTIC, Khalifa University of Science and Technology, Abu Dhabi, United Arab Emirates; 13 Department of Genetics and Molecular Biology, Khalifa University of Science and Technology, Abu Dhabi, United Arab Emirates; Jordan University of Science and Technology, JORDAN

## Abstract

Coronavirus disease 2019 (COVID-19) was first identified in respiratory samples and was found to commonly cause cough and pneumonia. However, non-respiratory symptoms including gastrointestinal disorders are also present and a big proportion of patients test positive for the virus in stools for a prolonged period. In this cross-sectional study, we investigated viral load trends in stools and nasopharyngeal swabs and their correlation with multiple demographic and clinical factors. The study included 211 laboratory-confirmed cases suffering from a mild form of the disease and completing their isolation period at a non-hospital center in the United Arab Emirates. Demographic and clinical information was collected by standardized questionnaire and from the medical records of the patient. Of the 211 participants, 25% tested negative in both sample types at the time of this study and 53% of the remaining patients had detectable viral RNA in their stools. A positive fecal viral test was associated with male gender, diarrhea as a symptom, and hospitalization during infection. A positive correlation was also observed between a delayed onset of symptoms and a positive stool test. Viral load in stools positively correlated with, being overweight, exercising, taking antibiotics in the last 3 months and blood type O. The viral load in nasopharyngeal swabs, on the other hand, was higher for blood type A, and rhesus positive (Rh factor). Regression analysis showed no correlation between the viral loads measured in stool and nasopharyngeal samples in any given patient. The results of this work highlight the factors associated with a higher viral count in each sample. It also shows the importance of stool sample analysis for the follow-up and diagnosis of recovering COVID-19 patients.

## 1. Introduction

COVID-19 is a severe acute respiratory tract infection caused by the novel Severe Acute Respiratory Syndrome Coronavirus 2 or SARS-CoV-2 [1]. The disease was first identified in December 2019, when several atypical pneumonia cases were reported in Wuhan City in China and rapidly spread worldwide [2, 3]. However, scientists are still investigating the exact origin, as evidence showed that SARS-CoV-2 virus was likely circulating for at least two months before the first identified cases [4]. As of October 1, 2021, the World Health Organization (WHO) had reported 233,503,524 confirmed cases, and 4,777,503 fatalities globally [5].

The majority of infected people have mild respiratory symptoms like fever, dry cough, and shortness of breath, as well as myalgia and fatigue [6]. Notably, since a large portion of the population worldwide is asymptomatic, it has been difficult to contain the spread of SARS-CoV-2 [7]. More severe symptoms can develop, namely pneumonia, among other complications as a result of the viral infection and can lead to death in susceptible groups [8]. Findings have shown a significant increase in cytokines in severe COVID-19 patients indicative of an unbalanced inflammatory response leading to a systemic inflammatory response, and multiple organ failure [2, 9]. SARS-CoV-2 can also cause gastrointestinal symptoms, such as vomiting, diarrhea, or abdominal pain, and affect multiple other organs [10]. The virus infects and replicates in host cells through binding of the viral Spike protein (S protein) with the Angiotensin-Converting Enzyme 2 (ACE2) cell surface receptor. ACE2 is expressed in various tissue types, including liver, stomach, ileum, kidney, heart, and colon [11–13], which explains the variety of symptoms observed in COVID-19 patients.

Infection control measures and the vast majority of research studies on SARS-CoV-2 focus on the respiratory system. However, reports have shown that up to 59% of patients test positive in stools and that the viral load in these samples can be higher than the viral load in pharyngeal swabs [14, 15]. Indeed, this fact has made it possible to monitor the virus at a population-level scale and to implement SARS-CoV-2 surveillance programs through the measurement of viral RNA in municipal wastewater in the US, Italy, and the United Arab Emirates [16–20]. This approach was efficient, especially since a proportion of asymptomatic patients who receive negative RT-PCR test results from the nasopharyngeal swabs still test positive for COVID-19 when stool-derived viral RNA was analyzed [6]. Studies have also shown that patients who recover and test negative for COVID-19 in nasopharyngeal swabs can still have positive stool test days after recovery [21]. However, the clinical significance of prolonged virus detection in stool samples is still unclear. The high viral loads and the prolonged detection of sub-genomic mRNA in stool samples strongly suggest an enteric infection of SARS-CoV-2 [15]. Few studies have even reported the isolation of infectious virus from patients’ stool [22–24]. Furthermore, recent studies have presented evidence on the prolonged detrimental alterations of the gut microbiome community in COVID-19 patients and their role in determining the severity of the infection and the duration of symptoms [25–27].

Principally, the knowledge about SARS-CoV-2 in the digestive tract is restricted to qualitative studies involving hospitalized and severe cases. Quantitative studies on the other hand are scarce and the factors associated with fecal viral loads have not been fully explored. This limits our understanding of the virus and makes disease control more challenging, especially with the emergence of new SARS-CoV-2 variants. In this perspective, we performed a cross-sectional study of 211 laboratory-confirmed COVID-19 cases to evaluate the correlation between the viral loads detected in stools versus nasopharyngeal swab samples and to explore the factors associated with the quantified viral titers in each of the tested samples.

## 2. Materials and methods

### 2.1. Study design and participants

A cross-sectional observational study with a single period of data collection for each participant was performed, recruiting a total of 211 participants who were staying at an isolation facility for COVID-19 patients in Abu Dhabi, United Arab Emirates (UAE) at the time of the study and when the samples were collected. Written informed consent for viral load monitoring was signed by all study participants before proceeding with sample collection. For participants under the age of 18, a parental or guardian consent form was obtained. The study was approved by the Abu Dhabi Health COVID-19 Research Ethics Committee (DOH/DQD/2020/538) and the Abu Dhabi Health Services Company (SEHA) Research Ethics committee (SEHA-IRB-005). Each participant filled out a questionnaire and provided metadata information on age, gender, COVID-19 symptoms, hospitalization, chronic diseases, smoking status, alcohol consumption, exercise frequency, and antibiotics and probiotics intake. The diagnosis date, SARS-CoV-2 vaccination status, blood type, weights, and heights of each participant were obtained from the medical records of the patient.

### 2.2. Sample collection

Samples were collected from different groups of patients on different dates: 44 samples in June 2020, 55 samples in July 2020, 44 samples in February 2021, and 68 samples in March 2021. Swabs were collected by nurses using Nucliswab kits (SALUBRLs, lnc. one Boston Place, suite 2600 Boston, MA 02108, USA). Plastic containers with spatula were given to the patients so they can provide a stool sample. Swab samples were transported at 4°C in an ice box to the laboratory of Khalifa University Centre for Biotechnology for testing, while the stool samples were stored in dry ice and transferred to the laboratory for testing.

### 2.3. SARS-CoV-2 viral RNA extraction and quantification

Viral RNA was extracted using the Miracle-AutoXT Automated Nucleic Acid Extraction System (iNtRON Biotechnology Inc, South Korea). The method consisted of transferring 200 μL of liquid from the nasopharyngeal swabs containers which contained a 3 mL aliquot of nucleic acid transport medium into prefilled deep well plates containing the manufacturer’s lysis, washing, and elution buffers. The internal RNA extraction control was added during the washing step for each sample to provide a template that allowed confirmation of successful extraction. For the stool samples, 200 mg of stool was diluted in 1 mL of 1X phosphate-buffered saline. The samples were vortexed to ensure homogeneity and 200 μL was used for the automated extraction using the method described above.

A Genesig® RT-PCR COVID-19 detection kit (Primerdesign Ltd, Watchmoor Point, UK) was used for the quantification of the viral RNA. The COVID-19 primers and probes (FAM channel) provided in the kit target the RNA-dependent RNA polymerase (RdRp) gene. Internal extraction control primers (VIC channel) were also provided for the detection of the exogenous source of RNA template added during the extraction step. The PCR reactions were performed according to the manufacturer’s instructions. Quantitative RT-PCR (qRT-PCR) was performed using the Magnetic Induction Cycler PCR Machine (Bio Molecular Systems, Queensland, Australia).

The qualitative detection of SARS-CoV-2 variants in positive cases (158 participants) was performed by RT-PCR assay using PowerChek SARS-CoV-2 S-gene mutation detection kit ver.1.0 (KOGENEBIOTECH, Seoul, Korea). In the case where the participant was positive for the virus in stools and nasopharyngeal swab, RNA extracted from nasopharyngeal swab was used as a template for this test. The assay detects key mutations or major variants of concern and variants of interest including N501Y, K417N, E484K, P681R, E484Q, and L425R, as well as the nucleocapsid (N) and RdRP genes to confirm sample positivity. The PCR reactions were performed according to the manufacturer’s instructions.

All PCR results were considered positive only when the cycle threshold value of the reference gene was <40.

### 2.4. Statistical analysis

Demographic and clinical data were descriptively analyzed and presented as median and range for continuous variables and as the number of participants and proportion (%) for categorical variables. Viral load values were log10-transformed for analysis and expressed as log10 RNA copies/mL. All categorical variables were expanded to multiple binary indicators using one-hot encoding, which essentially signifies the presence or absence of a specific value of the original variable. To calculate statistical significance between the variables and sample positivity, a two-sample independent t-test was used to detect the difference between groups for continuous variables. Categorical variables were compared by χ^2^ test or Fisher’s exact, and multiple z-tests as appropriate. All analyses were done with OriginPro 2020b (OriginLab Corporation, Northampton, MA, USA). Pearson’s linear correlation coefficient along with the two-sample independent t-test was used to measure the correlation and confirm/reject the null hypothesis of sample independence or the lack of linear relationship with the measured viral loads in nasopharyngeal swabs and stool samples. Missing data was handled by pairwise deletion. Statistical significance was defined as *p* < 0.05. All relevant data are available within the manuscript as well as a S1 Dataset.

## 3. Results

### 3.1. Baseline characteristics of participants

**Fig 1:**
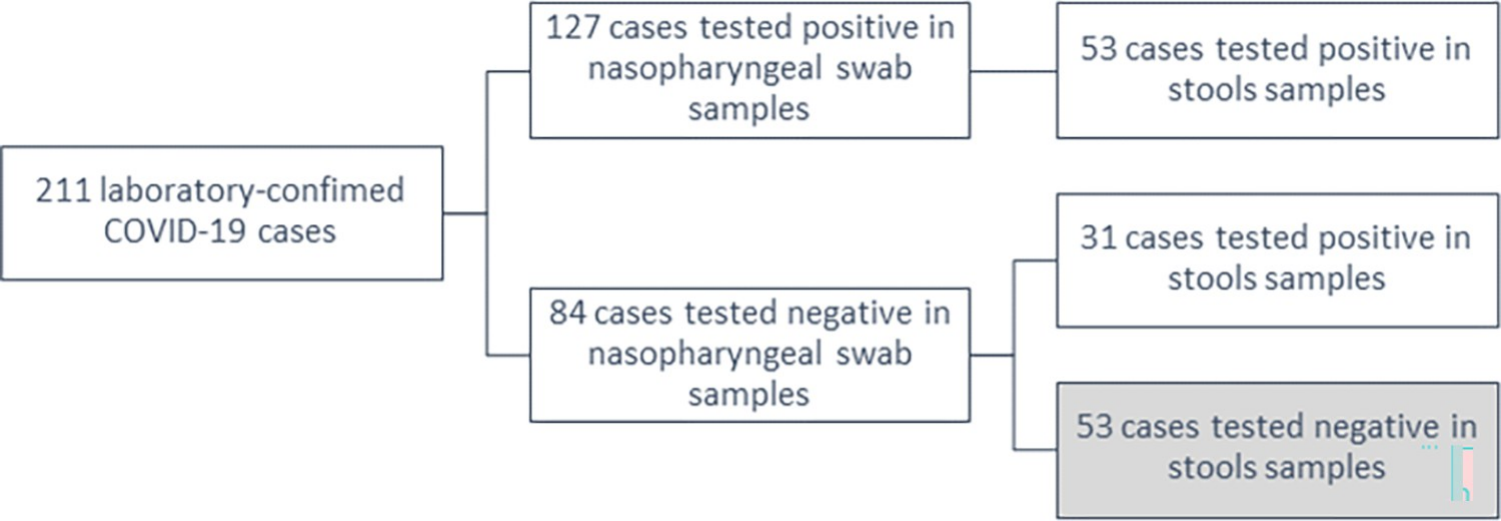
SARS-CoV-2 PCR test results among COVID-19 participants.

A total of 211 laboratory-confirmed COVID-19 cases were enrolled in this study between June 2020 and March 2021 (Fig 1). COVID-19 diagnosis was based on a positive RT-PCR test performed at a facility approved by SEHA. All participants suffered from a mild infection and were completing their isolation period at the time of this study in a nonhospital isolation facility. Participants were categorized as asymptomatic or mild cases according to the WHO criteria of classification [28].

Among enrolled participants, 127 (60%) cases tested positive in nasopharyngeal swabs at the time of the study. Of these patients, 53 (42%) cases tested also positive for SARS-CoV-2 in stools. In total, 31 (15%) participants tested positive only in stool samples and 53 (25%) cases showed a negative result for SARS-CoV-2 in both samples. The baseline characteristics of the participants by sample positivity are presented in Table 1.

**Table 1:** Baseline characteristics of the participants categorized by sample positivity.

	Total participants (n = 211) (%)	SARS-CoV-2 detected in stools only (n = 31) (%)	SARS-CoV-2 detected in nasopharyngeal swab only (n = 74) (%)	SARS-CoV-2 detected in stools and nasopharyngeal swab (n = 53) (%)	SARS-CoV-2 not detected (n = 53) (%)	*p*-value
**Median age (range)**						
	32 (13–69)	31 (16–69)	32 (18–69)	34 (13–56)	30 (17–68)	
**Gender**						
Male	164 (78%)	22 (71%)	23 (31%)	43 (81%)	37 (70%)	<0.001
**Symptoms**						
No symptoms	31 (15%)	6 (19%)	11 (15%)	5 (9%)	9 (17%)	
Loss of taste and smell	83 (39%)	10 (32%)	31 (42%)	21 (40%)	21 (40%)	
Diarrhea	55 (26%)	11 (35%)	12 (16%)	19 (36%)	13 (25%)	<0.001
Nausea	5 (2%)	0	0	5 (9%)	0	<0.001
Vomiting	16 (8%)	2 (7%)	6 (8%)	4 (8%)	4 (8%)	
Fatigue	74 (35%)	12 (38%)	30 (41%)	18 (34%)	14 (26%)	
Muscle or body aches	65 (31%)	9 (29%)	24 (32%)	14 (26%)	18 (34%)	
Headache	101 (48%)	14 (45%)	35 (47%)	30 (57%)	22 (42%)	
Fever	83 (39%)	12 (38%)	32 (43%)	25 (47%)	14 (26%)	
Runny or stuffy nose	54 (26%)	6 (19%)	16 (22%)	20 (38%)	12 (24%)	
Sneezing	39 (18%)	6 (19%)	14 (19%)	14 (26%)	5 (9%)	
Sore throat	58 (27%)	3 (10%)	20 (27%)	23 (44%)	12 (24%)	0.006
Shortness of breath	13 (6%)	0	7 (9%)	4 (8%)	2 (3%)	
Cough	90 (43%)	15 (48%)	30 (41%)	22 (42%)	23 (44%)	
Pneumonia	7 (3%)	2 (7%)	0	1 (2%)	4 (8%)	
**Symptoms required hospitalization**						
	17 (8%)	4 (13%)	1 (1%)	4 (8%)	8 (15%)	0.02
**Median duration of symptoms in days (range)**						
	4 (1–43)	4 (1–28)	3 (1–10)	3 (1–8)	4.5 (1–43)	0.02
**Median time between the onset of symptoms and study in days (range)**						
	6 (0–68)	11 (2–30)	6 (0–33)	6 (0–23)	8 (2–68)	0.01
**COVID-19 vaccination**						
Unvaccinated	165 (78%)	27 (87%)	55 (74%)	40 (75%)	43 (82%)	
First dose	7 (3%)	0	2 (3%)	3 (6%)	2 (3%)	
Second dose	39 (19%)	4 (13%)	17 (23%)	10 (19%)	8 (15%)	
**S protein mutation**						
No mutation^1^	46 (22%)	14 (45%)	15 (20%)	17 (32%)		
N501Y	40 (19%)	5 (16%)	18 (24%)	17 (32%)		
N501Y+K417N+E484K	12 (5%)	1 (3%)	8 (11%)	3 (5%)		
L452R	10 (4%)	0	8 (11%)	2 (3%)		
E484K	4 (2%)	0	3 (4%)	1 (2%)		
E484K+L452R	1 (0.5%)	0	1 (1%)	0		
P681R	1 (0.5%)	0	0	1 (2%)		
Not detected	44 (22%)	11 (36%)	21 (29%)	12 (24%)		
Not tested^2^	53 (25%)	0	0	0	53 (100%)	
**BMI (kg/m** ^**2**^ **)**						
	26.7 (16.4–46.9)	27.1 (16.4–37.2)	26.1 (17.9–46.9)	28.4 (18.5–40.1)	25.95 (18.2–36.5)	0.01
**Blood type**						
A	21 (10%)	5 (16%)	10 (13%)	2 (3%)	4 (8%)	
B	21 (10%)	2 (7%)	13 (18%)	3 (6%)	3 (6%)	
AB	9 (4%)	0	2 (3%)	3 (6%)	4 (8%)	
O	78 (37%)	10 (32%)	27 (36%)	25 (47%)	16 (29%)	
Unknown	82 (39%)	14 (45%)	22 (30%)	20 (38%)	26 (49%)	
**Rhesus factor**						
Negative	7 (3%)	1 (3%)	4 (5%)	0	2 (3%)	
Positive	98 (46%)	14 (45%)	33 (45%)	28 (53%)	23 (44%)	
Unknown	106 (51%)	16 (52%)	37 (50%)	25 (47%)	28 (53%)	
**Exercise**						
No	22 (10%)	3 (10%)	5 (7%)	7 (13%)	7 (13%)	
Occasionally	22 (10%)	8 (26%)	8 (11%)	3 (6%)	3 (6%)	
Regularly	51 (24%)	8 (26%)	10 (13%)	15 (28%)	18 (34%)	
Unknown	116 (56%)	12 (38%)	51 (69%)	28 (53%)	25 (47%)	
**Smoker**						
	68 (32%)	11 (35%)	24 (32%)	22 (42%)	11 (21%)	
**Consume alcohol **						
	14 (7%)	2 (7%)	6 (8%)	4 (8%)	2 (3%)	
**Chronic diseases**						
Diabetes	7 (3%)	2 (7%)	2 (3%)	2 (3%)	1 (2%)	
**Antibiotics treatment in the last 3 months**						
	19 (9%)	4 (13%)	6 (8%)	5 (9%)	4 (8%)	
**Probiotics intake**						
	20 (9%)	3 (10%)	7 (9%)	6 (11%)	4 (8%)	

Data are presented as the number of participants and proportion (%) for categorical variables and as median and range for continuous variables. BMI: body mass index.

^1^ Cases that showed a positive result for the N and RdRP genes and a negative result for the investigated mutations.

^2^ Samples that were negative for SARS-CoV-2 (stools and nasopharyngeal swab) at the time of the study were not tested for the variants (53 participants).

The median age of the participants was 32 years and 78% were males. Less males tested positive for SARS-CoV-2 in nasopharyngeal swabs alone compared to in stools and in both samples (*p* < 0.001). Thirty-nine participants (19%) were fully vaccinated (2 vaccine doses) against SARS-CoV-2 at the time of this study. There were 31 asymptomatic cases (15%), and the reported symptoms in the case of symptomatic patients included uncomplicated upper and lower respiratory illnesses and digestive disorders. The predominant symptoms reported by more than 40% of the participants included headache, and cough while the least reported symptoms (<10%) included pneumonia, shortness of breath, nausea, and vomiting. Combined gastrointestinal symptoms were reported by 36% of the patients with diarrhea being the most common (26%). Cases that tested positive in stools only were more likely to present with diarrhea (*p* = 0.03) and to be admitted to the hospital during the infection (*p* = 0.01) compared to nasopharyngeal swab-positive patients.

Symptoms lasted for a median duration of 4 days. Participants who tested negative for SARS-CoV-2 reported a longer duration of symptoms compared to nasopharyngeal swab-positive cases (*p* = 0.04). The duration of symptoms correlated with male gender *r*(209) = -0.45, *p* < 0.001, absence of vaccination *r*(209) = 0.21, *p* = 0.002, two doses of vaccine received *r*(209) = -0.18, *p* = 0.01, hospitalization *r*(209) = 0.19, *p* = 0.005, blood type B *r*(209) = -0.22, *p* = 0.001, rhesus negative *r*(209) = 0.47, *p* < 0.001, and a number of reported symptoms (S1 Table). The median time between the onset of symptoms and a positive RT-PCR test in this study was 6 days. This duration was longer (11 days) for stools-positive cases compared to nasopharyngeal swab-positive patients (*p* = 0.02) and shorter for swab-positive cases compared to participants that tested negative in both samples (*p* = 0.01). Interestingly, out of the 22 patients who reported the onset of symptoms at least 15 days before this study, 54% (n = 12) were stool-positive cases and 18% (n = 4) had detectable SARS-CoV-2 in nasopharyngeal swabs.

The median Body Mass Index (BMI) of the participants was 26.7 kg/m^2^. BMI was higher for participants that tested positive for the virus in both samples compared to swab-positive only (*p* = 0.04) and negative cases (*p* = 0.03). The predominant blood type was O accounting for 37% of the participants and 46% were rhesus positive. Active smokers comprised 32% of the cases and 24% reported exercising regularly. Only 7 participants (3%) suffered from diabetes and the other cases did not report any chronic disease. Among the cases, 9% reported taking antibiotics in the last 3 months, 9% previously took probiotics, and 93% did not consume alcohol.

**Fig 2:**
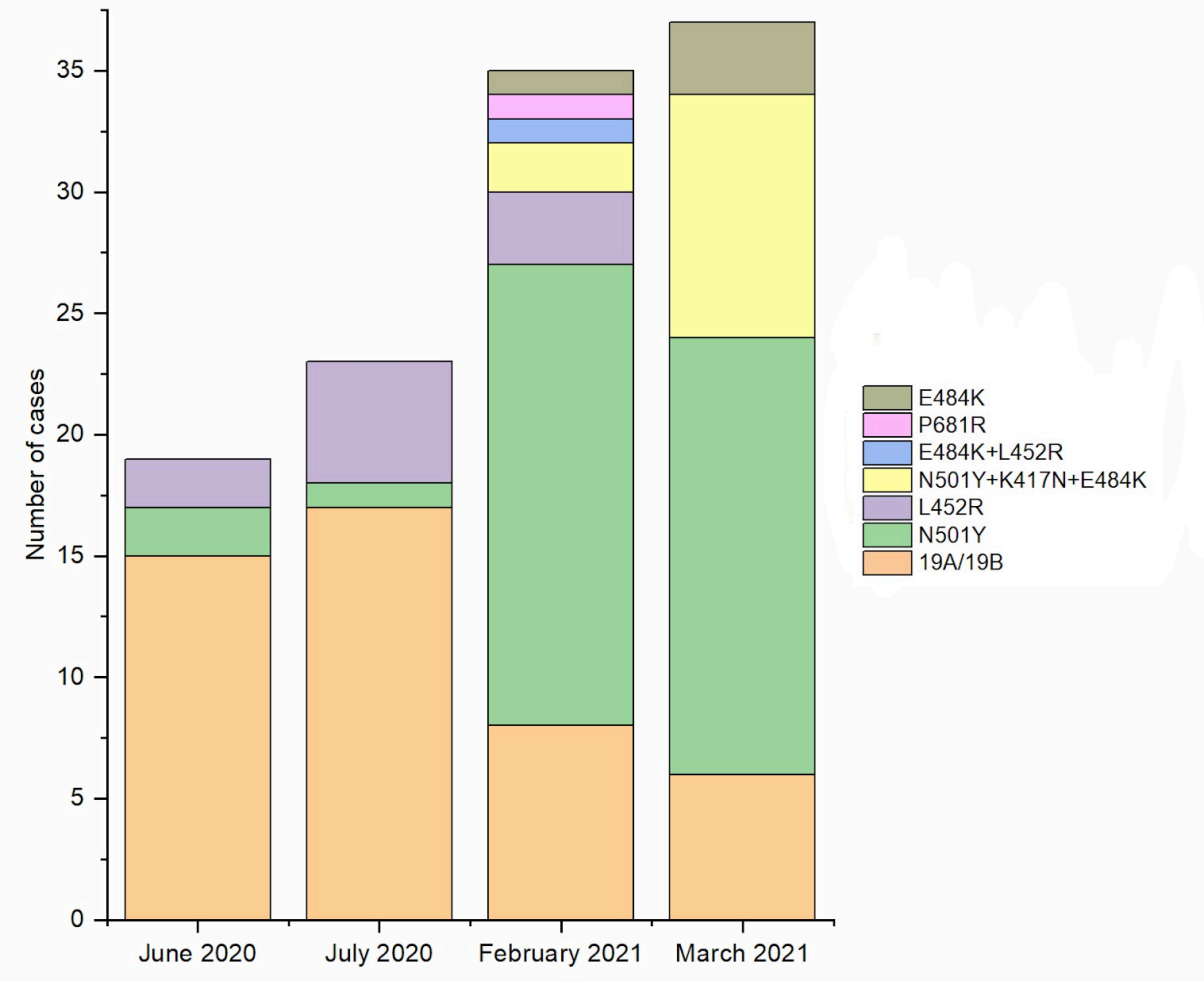
The distribution of SARS-CoV-2 S protein mutations detected in samples collected during four different periods between 2020 and 2021.

The predominant SARS-CoV-2 strain was 19A/19B (46%) and no association between the detected mutations and the positivity of samples was observed. The temporal distribution of the detected mutations among the participants showed that samples collected during June and July 2020 had mainly no detectable amino acid substitution mutation in the S protein region (Fig 2). Meanwhile, samples collected in 2021 showed a higher proportion of the N501Y, K417N and E484K mutation associated with the B.1.1.7 and B.1.351 variants.

### 3.2. SARS-CoV-2 viral loads in stools and nasopharyngeal swabs

**Fig 3:**
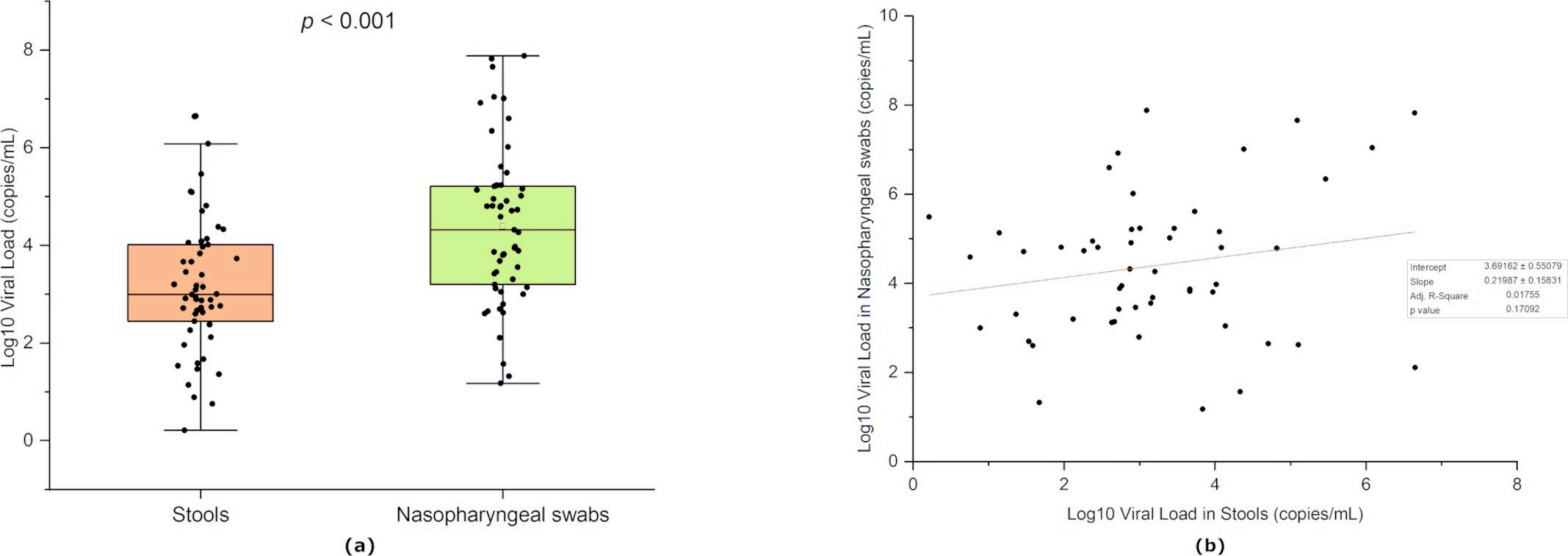
Comparison between viral loads in stools and nasopharyngeal swabs. (**a**) Comparison of the viral load in stools and nasopharyngeal swabs of cases that tested positive in both samples; (**b**) linear regression analysis of the log10 viral load for each case in stools (X-axis) against the log10 viral load in nasopharyngeal swab (Y-axis). The graph presents the median and the minimal to the maximal range of viral load in log10 copies/mL. The significance of the pairwise two-sided t-tests is indicated on top.

Quantitative RT-PCR was performed to quantify SARS-CoV-2 viral RNA copies in stools and nasopharyngeal swab samples. The quantified viral loads in the nasopharyngeal swabs of participants that tested positive for the virus in both samples were significantly higher than that detected in stool samples (*p* < 0.001) (Fig 3A). The viral loads ranged from 1.63 and 4.45×10^6^ copies/mL, with a median of 1.01×10^3^ copies/mL in stools and between 15 to 1.78×10^8^ copies/mL, with a median of 4.79×10^3^ copies/mL in nasopharyngeal swabs. Regression analysis showed no correlation between the viral loads measured in stool and nasopharyngeal samples in a given patient *R*^*2*^ = 0.01, *F*(1, 52) = 1.92, *p* = 0.17 (Fig 3B).

**Fig 4:**
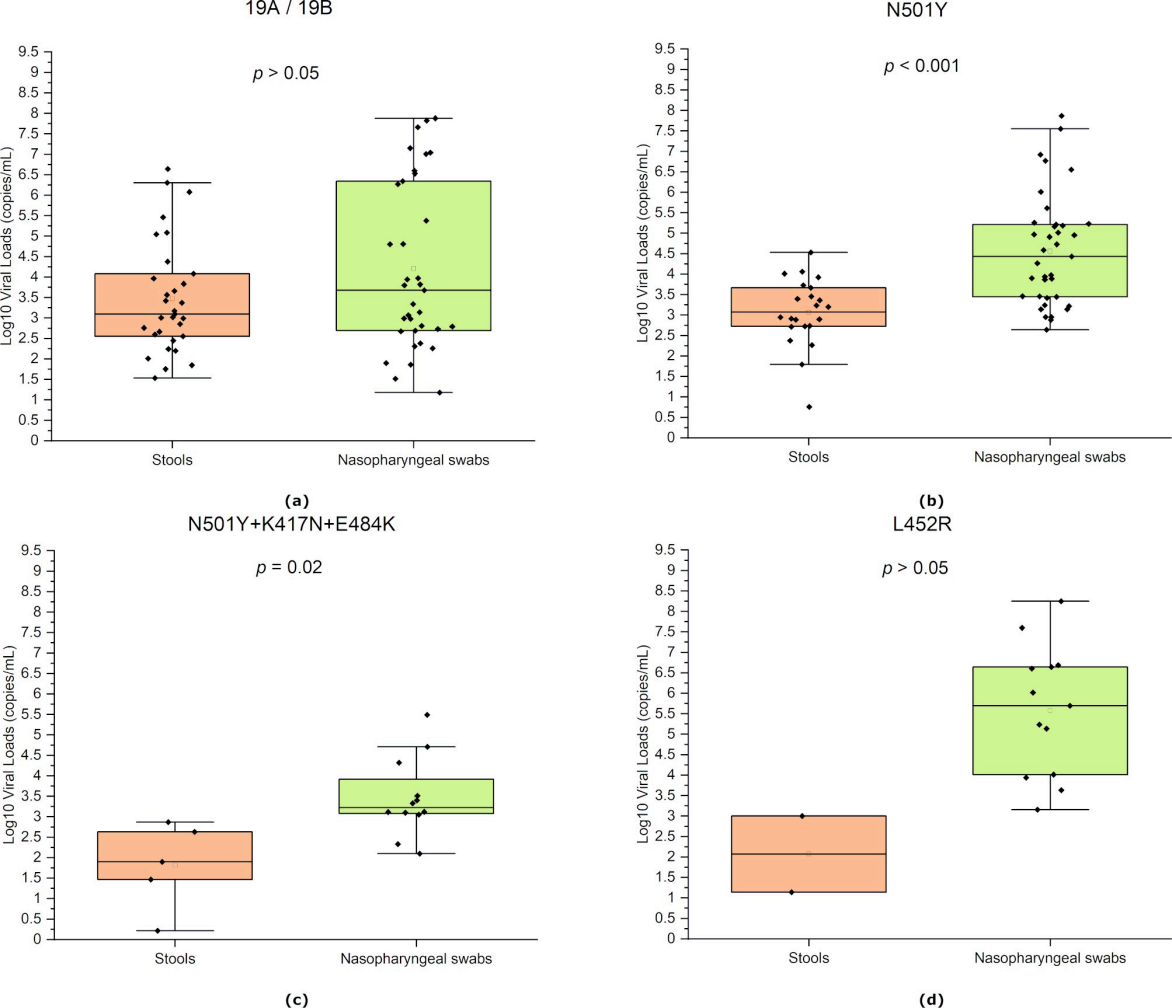
Comparing viral loads in stools and nasopharyngeal swabs of patients infected by different SARS-CoV-2 variants. (a) negative for S protein mutations (b) N501Y mutation, (c) N501Y+K417N+E484K mutations, and (d) L452R mutation. The graph presents the median and the minimal to the maximal range of viral load in log10 copies/mL. The significance of the pairwise two-sided t-tests is indicated on top.

An additional analysis comparing the viral load in stools and nasopharyngeal swabs between the four most identified S protein mutations in the studied group (≥10 cases) was performed. The quantified viral load was significantly higher in the nasopharyngeal swabs compared to stools for N501Y mutation (*p* < 0.001), and N501Y+K417N+E484K mutations (*p* = 0.02) (Fig 4).

The viral load in nasopharyngeal swabs from patients testing positive for the L452R mutation was significantly higher compared to strain 19A / 19B (*p* = 0.02), and mutations N501Y+K417N+E484K (*p* = 0.008). On the other hand, the viral loads in stools were significantly higher in the case of SARS-CoV-2 19A / 19B compared to N501Y+K417N+E484K mutations (*p* = 0.04).

**Fig 5:**
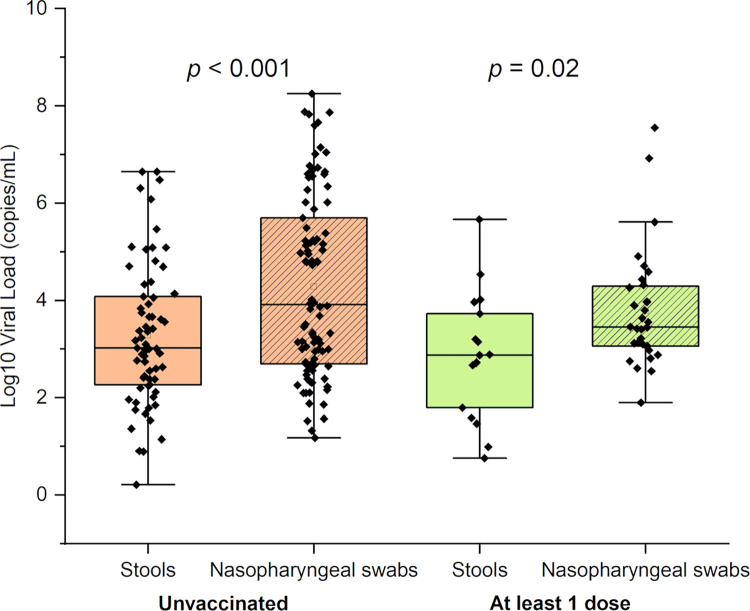
Viral load in stools and nasopharyngeal swabs of unvaccinated and vaccinated (at least 1 dose) participants. The graph presents the median and the minimal to the maximal range of viral load in log10 copies/mL. The significance of the pairwise two-sided t-tests is indicated on top.

The viral loads in samples taken from unvaccinated and vaccinated (at least 1 dose) participants were also compared. The viral load in nasopharyngeal swabs was significantly higher than that detected in stools for both, unvaccinated (*p* <0.001) and vaccinated individuals (*p* = 0.02) (Fig 5).

### 3.3. Factors affecting viral shedding in stools and nasopharyngeal swabs

The demographic and clinical factors and their correlation with the viral load of SARS-CoV-2 in either stool samples or nasopharyngeal swabs are presented in Tables 2 and 3, respectively.

**Table 2:** Factors associated with changes in viral load in stools.

	95% CI	*p*-value	Coefficient (*r*)
**Gender**			
Male	-0.68, -0.09	0.02	-0.43
**Symptoms**			
Nausea	0.62, 0.83	<0.001	0.74
**Hospitalization**			
	0.04, 0.54	0.02	0.31
**Blood type**			
O	-0.001, 0.34	0.05	0.17
**BMI**			
Overweight	0.002, 0.41	0.05	0.22
**Exercise**			
Regular	-0.01, 0.54	0.05	0.29
**Antibiotic treatment in the last 3 months**			
	<0.001, 0.56	0.05	0.31

**Table 3:** Factors associated with changes in viral load in nasopharyngeal swabs.

	95% CI	*p*-value	Coefficient (*r*)
**Symptoms**			
Diarrhea	0.04, 0.54	0.02	0.32
**Blood type**			
A	0.20, 0.64	<0.001	0.45
**Rhesus**			
Positive	0.03, 0.36	0.02	0.20
**Antibiotic treatment in the last 3 months**			
	0.009, 0.57	0.04	0.32
**Probiotics intake**			
	0.10, 0.63	0.01	0.40

The correlation analysis in patients that tested positive for the virus in stools and nasopharyngeal swabs indicated a positive correlation between the viral load in this sample and nausea (*r*(51) = 0.74, *p* <0.001), hospitalization during COVID-19 infection (*r*(51) = 0.30, *p* = 0.0), and blood type O (*r*(51) = 0.17, *p* = 0.05). In cases that tested positive in stools and either positive or negative in nasopharyngeal swabs, higher viral load in stools correlated with being overweight (*r*(82) = 0.22, *p* = 0.05), exercising (*r*(82) = 0.29, *p* = 0.05), and taking antibiotic treatment in the last 3 months (*r*(82) = 0.31, *p* = 0.05). In participants that tested positive for the virus only in stools, males showed a significantly lower viral load in this sample compared to females (*r*(29) = -0.43, *p* = 0.02).

On the other hand, viral load in nasopharyngeal swabs positively correlated with diarrhea as symptom (*r*(51) = 0.32, *p* = 0.02), blood type A (*r*(51) = 0.45, *p* < 0.001), and probiotics intake (*r*(51) = 0.40, *p* = 0.01) in patients who tested positive for the virus in stools and nasopharyngeal swabs. Higher viral load in all nasopharyngeal swab-positive cases positively correlated with rhesus positive (*r*(125) = 0.20, *p* = 0.02), antibiotic treatment in the last 3 months (*r*(125) = 0.32, *p* = 0.04), and probiotics intake (*r*(125) = 0.40, *p* = 0.01).

## 4. Discussion

Samples in this study were collected at two different periods; June/July 2020 and February/March 2021, shortly after the two major peaks in cases were reported in the UAE [29]. The first batch of samples was collected in 2020 while the second batch was collected in 2021, two months after the Ministry of Health and Prevention had announced the vaccine was available for all UAE citizens and residents aged 16 and above [30]. By collecting samples in 2020 and 2021, we were able to include two important variables in the analysis: vaccination and SARS-CoV-2 variant.

The results obtained in this study showed that 40% of mild laboratory-confirmed COVID-19 patients tested positive for viral RNA in stool samples, which was similarly reported by other groups. Chen et al. (2020) reported the detection of SARS-CoV-2 in stool specimens of 67% of patients, with 64% of them maintaining a positive PCR result in the stools even after PCR result from the pharyngeal swabs turned negative [21]. Studies investigating the time frame of viral shedding in feces have shown an extended duration of viral detection in stool samples compared to nasopharyngeal swabs. Nasopharyngeal swabs remained positive for a mean of 15.4 days while fecal samples remained positive for a mean of 27.9 days after the first symptom onset [31]. In addition, stool samples remained positive between 1 to 33 days after the nasopharyngeal swab turned negative [32]. Even though in our study, the viral loads in both samples were not followed over time, 15% of the patients tested positive in stools after their nasopharyngeal swab test turned negative. In addition, the median time between the onset of symptoms and the RT-PCR test performed in this study was longer for cases that tested positive in stools only (11 days) compared to those that tested positive in nasopharyngeal swabs (6 days), which is in agreement with previous findings reporting that viral load in stools peaks later during infection [23].

Although the participants in this study were mild cases with 15% showing no symptoms at all, the viral copies detected in the stools and nasopharyngeal swabs reached 4.45×10^6^ and 1.78×10^8^ genome copies/mL, respectively, which is comparable to those reported in hospitalized cases [33, 34]. The similarity between the viral loads detected in asymptomatic and symptomatic patients was previously highlighted by Zou et al. (2020) [35]. We also observed higher viral titers in respiratory samples compared to stool samples in patients that tested positive in both samples, which mirrors observations reported previously [14]. However, no correlation was observed between the measured viral load in stool and nasopharyngeal swab in a given patient. This suggests the possibility of different virus incubation periods for respiratory and enteric infections, a differing rate of viral replication in each system, and/or a different rate of viral shedding. This is supported by the previously observed correlation between the viral loads in different respiratory specimens (nasopharyngeal, oropharyngeal, and sputum) [36]. However, in the absence of follow-up of viral loads in each sample, it is difficult to generalize the observed difference in titers and the absence of correlation to the entire period of infection.

Interestingly, the same difference in viral loads between samples was observed for the wild-type SARS-CoV-2 strains, B.1.1.7 (N501Y), and B.1.351 (N501Y+K417N+E484K) variants. Mutation L452R was associated with the highest viral count in nasopharyngeal swabs compared to other variants, whereas cases infected by strains 19A/19B of the virus had a higher viral load in stools compared to cases infected by variant B.1.351. Emerging SARS-CoV-2 variants accumulate mutations in the S protein region, resulting in altered interactions with the host [37], which could possibly explain the variations in the viral load detected in stools of patients infected by SARS-CoV-2 variants. These mutations have been found in some cases to trigger higher transmission and virulence [38], and potential resistance to natural or acquired immunity [39, 40], and this can be linked to a higher replication rate of the virus. Indeed, the L452R mutation is characteristic of SARS-CoV-2 variant B.1.427/429, which has shown 4 times higher infectious units per quantity of viral E gene RNA compared to the Alpha variant [41].

The temporal distribution of the detected mutations across the different sampling-period groups showed that the wild-type strains were predominant in 2020. In February and March 2021, a higher proportion of N501Y, K417N, and E848K mutations was detected, which can be related to the global peak in Alpha and Beta variant cases during that period. Very few samples from June and July 2020 tested positive for the N501Y and L452R mutations, suggesting that these mutations have been circulating at the early stages of the outbreak before mutants harboring these mutations were first collected and described [42, 43]. Indeed, L452R mutation was first detected in the B.1.39 lineage in Denmark on March 17, 2020 (GISAID ID: EPI_ISL_429311), and the oldest sequence harboring this mutation in the B.1.427/B.1.429 lineage was isolated in Mexico, on July 6, 2020 (GISAID ID: EPI_ISL_942929) [44]. The spatial and temporal distribution of SARS-CoV-2 variants in the UAE is under-reported. Two studies have shown a wide diversity in the introduced SARS-CoV-2 strains into the UAE due to the country being multinational and a major international travel hub in the Middle East [45, 46]. The Beta variant was detected in SARS-CoV-2 positive individuals who traveled from UAE to India during February–March 2021, suggesting that this variant was circulating in the UAE at the time [47]. Interestingly, a phylogeny analysis on 69 SARS-CoV-2 genome sequences collected from COVID-19 patients in the UAE, showed that 1 sample belonged to lineage B.1.1.7, confirming the early presence of the alpha variant in the population during April-July 2020 [48].

When the viral loads of unvaccinated and vaccinated (at least 1 dose) participants were compared, no significant difference was observed in stools and nasopharyngeal swabs between the two groups. The primary administered vaccine in the Emirate of Abu Dhabi at the time of this study was the inactivated whole virus vaccine produced by Sinopharm. Early studies on this vaccine demonstrated a significant decrease in viral load in the throat of macaques 10 days after second immunization compared to the placebo group [49], yet there is no information available on infection in vaccinated humans. It is also difficult to draw conclusions as samples in this study were collected during the early stage of the UAE COVID-19 vaccination campaign, and therefore many immunized subjects were not considered “effectively immunized” (defined as 28 days after administration of the second dose).

Previous studies have shown that fever and cough were the main symptoms reported by patients. In an early study conducted between January 2020 and February 2020 on 96 COVID-19 patients, 89% of the cases developed fever and 56% reported coughing [14, 50]. Similarly, in our study, 43% of the participants reported having a cough. However, fever was less common (39%) compared to previous studies which is possibly due to participants being mild cases that did not require hospitalization. The most common symptom reported in our study was headache (48%).

Diarrhea was the major described non-respiratory symptom (26%), and this symptom was significantly more reported in patients that tested positive for the virus in stools. The same observation was previously reported in several small case series and large case studies reviewed in [51]. However, no correlation between the reported enteric symptoms andthe quantified viral loads in stools was observed. Recent studies have shown that the prevalence of enteric symptoms can be as high as 50% [52]. However, data related to gastrointestinal disorders is very prone to confounding as such symptoms are very often underreported [53], sometimes associated with the intake of medication [54], or even triggered by anxiety [55].

A positive correlation was also found between recent antibiotic intake and the quantified viral loads in stools and nasopharyngeal swabs. The intake of antibiotics has shown a significant compositional disturbance and reduced diversity in the gut microbiome [56]. The association between COVID-19 severity and the composition of the gut microbiome was recently investigated and significant alterations in bacteria from the phyla Bacteroidetes and Firmicutes were observed in COVID-19 patients [57]. These bacteria have been shown to control ACE2 expression in murine gut [58] modulating human gut response to SARS-CoV-2 infection and affecting the viral load in stools. The gut microbiome is also known to regulate the pulmonary immune response against viral respiratory infections through the gut–lung axis [59], possibly explaining the higher viral load in respiratory specimens after antibiotics treatment and probiotics intake.

Differences in the susceptibility and response of different populations to the COVID-19 pandemic can be highly affected by differential genetic factors. The distribution of blood types for example significantly varies among populations. In this study, 30% of the participants had blood group O-positive which reflects the wide distribution of this blood type among the UAE population [60]. A positive correlation was observed between the viral load in nasopharyngeal swabs and blood type A and rhesus positive. Most studies have suggested that people with blood type A have a higher risk and people with blood type O have a lower risk of COVID-19 infection [61, 62]. Testing positive for SARS-CoV-2 has also been found to correlate with rhesus-positive [63]. The proposed underlying mechanism is a protective effect of the IgG anti-A/B in group O plasma by binding to the S protein of the virus and reducing or preventing the interaction with the ACE2 receptor [64, 65].

In addition to genetic differences, dietary and lifestyle habits can also shape the outcome of COVID-19 infection. In previous studies, obesity was a major risk factor for severe COVID-19 infection [66]. However, the relationship between BMI and SARS-CoV-2 viral shedding has yet to be described. A significant correlation between the viral loads in stool samples and being overweight was observed in this study. In addition, participants that tested negative in both samples had significantly lower BMI than cases testing positive in both. ACE2 is largely expressed in visceral adipose tissue possibly contributing to the higher viral loads in overweight patients [67]. In addition, higher weight is characterized by an increased gut permeability and microbial dysbiosis possibly rendering the host’s digestive system more susceptible to SARS-CoV-2 replication. A significant correlation was also observed between the viral load in stool samples and exercising. Studies conducted on mice showed that exercising significantly increased IL-4 lung protein levels which is involved in initiating the production of antibodies against viruses leading to reduced lung inflammation during influenza infection [68]. A boost in lung defense mechanisms includes an optimal ciliary function through mucociliary clearance of apically released virus which then gain access to the gastrointestinal tract. SARS-CoV and SARS-CoV-2 infection downregulate ACE2 expression resulting in the overactivation of Angiotensin II, which induces a strong inflammatory response and multiorgan dysfunction [69, 70]. The balance of the Renin-Angiotensin System (RAS) components plays a crucial role in determining the outcome of the infection. Physical exercise produces a shift of RAS balance toward ACE2, Angiotensin-(1–7), and Mas receptor, which is involved in vasodilation, anti-inflammation and cardioprotective actions [71].

This study has several limitations. It focused on asymptomatic and mild patients which are completing the isolation period in non-hospital facilities. These cases are generally younger, have no to mild symptoms and possibly lower viral loads, and therefore do not represent all COVID-19 patients. As the participants in this study were all in isolation facilities and not hospitals, self-reporting was the main method for collecting information which implicates uncertainties about some of the communicated details. The extraction of good-quality RNA from stool samples is also challenging due to the complex nature of of the sample.

## 5. Conclusion

Overall, in this study, 42% of participants that tested positive for the virus in nasopharyngeal swabs also tested positive in stools highlighting the high rate of viral shedding in stools among COVID-19 patients and demonstrating that wastewater-based epidemiology could provide estimates of infectious cases in the population. Based on the results reported in this study, wastewater-based surveillance has the advantage of monitoring virus shedding over time from symptomatic, asymptomatic, pre-symptomatic, and post-symptomatic individuals. This approach can present valuable information on the prevalence of the virus in the community and the early detection of outbreaks. Previously, studies investigating SARS-CoV-2 detection in stool samples were qualitative and involved hospitalized and severe cases. In this study, we explored the quantitative pattern of viral shedding in mild cases and highlighted the disparity between viral shedding in stools and nasopharyngeal swabs. In addition, we identified for the first time factors associated with changes in the quantified viral load in stool sample. While respiratory shedding is associated with early infection and higher transmission, analyzing fecal specimens can play an important role in the control and follow-up of COVID-19 infection, especially during recovery. This work also underlines the necessity of personalized therapy that takes into consideration the variances in susceptibilities of different organs based on genetic and physical markers and the complex relationship between the Microbiome Signature and the immune system during viral infections.

## Supporting information

S1 Dataset Minimal data set. (XLSX)

S1 Table Factors associated with changes in the duration of symptoms. (DOCX)
