# Supplementary material for: Analysis of SARS-CoV-2 viral loads in stool samples and nasopharyngeal swabs from COVID-19 patients in the United Arab Emirates
Source: PLoS One. 2022 Sep 22;17(9):e0274961. doi: 10.1371/journal.pone.0274961 (PMC9499247; doi:10.1371/journal.pone.0274961)
Supplement: S1 Table — (DOCX) [file pone.0274961.s002.docx]

**Table S1. Factors associated with changes in the duration of symptoms.**

|  | **95% CI** | ***p*-value** | **Coefficient (*r*)** |
| --- | --- | --- | --- |
| **Gender** | | | |
| Male | -0.55, -0.34 | < 0.001 | -0.45 |
| **Symptoms** | | | |
| Muscle/Body ache | 0.06, 0.33 | 0.003 | 0.20 |
| Vomiting | 0.09, 0.35 | < 0.001 | 0.23 |
| Nausea | 0.07, 0.34 | 0.002 | 0.21 |
| Cough | 0.06, 0.33 | 0.003 | 0.20 |
| Diarrhea | 0.02, 0.29 | 0.02 | 0.16 |
| **Time between the onset of symptoms and study** | | | |
|  | 0.56, 0.72 | < 0.001 | 0.65 |
| **COVID-19 vaccination** | | | |
| No vaccination | 0.07, 0.34 | 0.002 | 0.21 |
| Two doses received | -0.31, -0.04 | 0.01 | -0.18 |
| **Hospitalization** | | | |
|  | 0.05, 0.32 | 0.005 | 0.19 |
| **Blood type** | | | |
| B | -0.34, -0.08 | 0.001 | -0.22 |
| **Rhesus factor** | | | |
| Negative | 0.36, 0.57 | < 0.001 | 0.47 |
